# Exosome-mediated tendon-derived stem cell therapy strategies: potential and challenges

**DOI:** 10.3389/fbioe.2026.1822635

**Published:** 2026-06-17

**Authors:** Yuke Chen, Xin Li, SongOu Zhang, Xujun Hu

**Affiliations:** 1 School of medicine, Shaoxing University, Shaoxing, Zhejiang, China; 2 School of medicine, Ningbo University, Ningbo, Zhejiang, China; 3 Department of Orthopedics, Shaoxing People’s Hospital, Shaoxing People’s Hospital Affiliated to Shaoxing University, Shaoxing, Zhejiang, China

**Keywords:** exosomes, intercellular communication, TDSCs, tendon diseases, tendon repair

## Abstract

Tendon injuries are common disorders of the musculoskeletal system, and current treatments exhibit significant limitations in promoting effective regeneration. Recently, cell-based regenerative medicine has offered promising strategies for tendon repair. Among these, tendon-derived stem cells (TDSCs) have attracted substantial attention due to their robust self-renewal capacity and multilineage differentiation potential. Cell-free stem cell therapies, particularly those mediated by exosomes derived from stem cells, have emerged with significant therapeutic potential. This review summarizes the fundamental characteristics of TDSCs and their contributions to tendon repair, highlighting the pivotal roles of exosomes as critical mediators of intercellular communication. Furthermore, it outlines current methods for isolating and characterizing exosomes derived from TDSCs, evaluates their biological functions, and examines their therapeutic efficacy in animal models, emphasizing prospects for future clinical translation. Finally, this article addresses the advantages of exosome-based therapies regarding safety and efficacy, while discussing existing challenges and future perspectives in large-scale exosome production, quality control, and in-depth exploration of underlying mechanisms. Exosome-mediated TDSCs therapy represents a promising, emerging therapeutic approach.

## 1 Introduction

**FIGURE 1:**
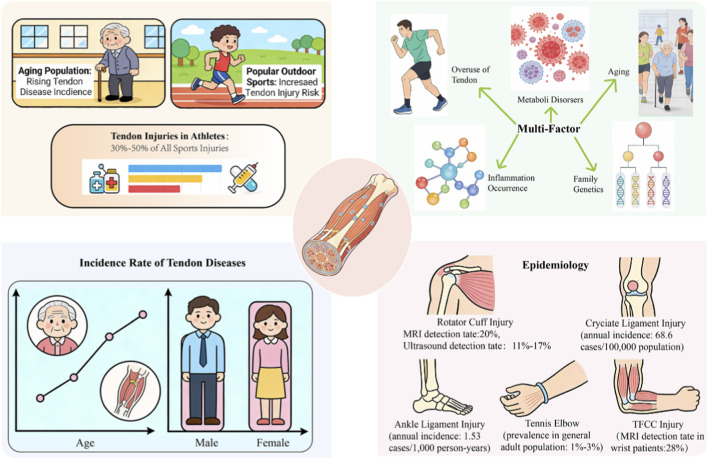
Basic Information on Tendon Diseases. This figure outlines the clinical burden, epidemiological features, and multifactorial pathogenesis of tendon diseases. It shows rising incidence driven by aging and sports participation, age- and gender-dependent prevalence trends, and key risk factors including overuse, aging, metabolic disorders, inflammation, and genetics. Epidemiological data for common tendon/ligament injuries and a tendon anatomy schematic are also provided.

Tendinopathy is a highly prevalent musculoskeletal disorder characterized by pain, functional impairment, and reduced exercise tolerance (Millar et al., 2021). It severely impairs patients’ quality of life and imposes a substantial socioeconomic healthcare burden. With population aging and the popularization of national fitness initiatives, the incidence of tendon injuries and degenerative lesions continues to rise. Approximately 2% of the general population develops lower-extremity tendinopathy before the age of 65 (Linsell et al., 2006), while such conditions account for 30%–50% of all sports-related injuries (Järvinen et al., 2005; Sharma and Maffulli, 2005). Tendinopathy has therefore become an urgent challenge to be addressed in sports medicine and regenerative medicine. The pathogenesis of tendinopathy arises from the combined effects of multiple contributing factors (Maffulli et al., 2023).Epidemiological studies on the impact of sex and age factors revealed that the incidence of tendon disorders increases with increasing age. It appears that women might be at a slightly higher risk of developing tendon disorders compared to men (GBD 2021 Other Musculoskeletal Disorders Collaborators, 2023; Kwan et al., 2023; Hefferan et al., 2025). Tendon disorders comprise tendon injuries and tendinopathy. Tendon injuries typically involve degeneration due to repeated mechanical stress from high-intensity physical activities (Millar et al., 2021). These representative conditions include rotator cuff tears, cruciate ligament injuries, ligament sprains around the ankle, triangular fibrocartilage complex injuries, and lateral epicondylitis, which is commonly known as tennis elbow. Tendinopathy refers to tendon pain often linked with inflammation, including conditions such as tendinitis, peritendinitis, and combined peritendinitis with tendinitis (Maffulli et al., 2003). Surgical treatment represents a major treatment modality for tendon disorders. However, the postoperative results often prove to be unsatisfactory. For the treatment of refractory tendon disorders, surgery sometimes proves to be ineffective (Deng et al., 2025). Despite the advancement of surgical techniques, the exact pathophysiological mechanisms of tendon disorders remain poorly understood. Consequently, it is imperative to understand that the current therapeutic approaches have limitations, thus calling for innovative approaches. In recent years, TDSC-derived exosomes have become a research hotspot in tendon regeneration therapy due to their inheritance of the biological activity of the parent cells and the avoidance of risks associated with cell transplantation. This review discusses the role of TDSCs in tendon healing, advances in exosome research, and the therapeutic potential and challenges of TDSC-derived exosomes (Figure 1).

## 2 TDSCs: introduction and their significance in tendon disorder treatment

### 2.1 Basic characteristics of TDSCs

**FIGURE 2:**
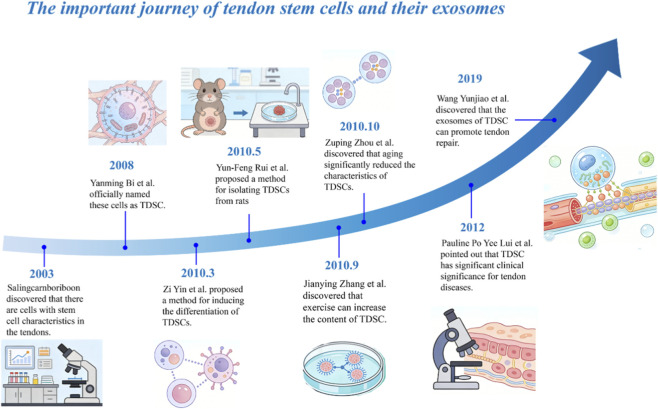
Research History of TDSCs. This timeline figure systematically traces the landmark research milestones in the field of tendon-derived stem cells (TDSCs) and their derived exosomes, from the initial discovery of tendon-resident stem cells to the exploration of exosome-based regenerative therapies for tendon repair. Collectively, this timeline illustrates the progressive evolution of TDSC research, from basic cell characterization to the exploration of exosome-based therapies, and contextualizes the core focus of the present review: the therapeutic potential of TDSC-derived exosomes in tendon repair.

TDSCs can be isolated from tendon tissues of different animal species. TDSCs were first identified from tendons of humans and mice by Salingcarnboriboon and Bi and later identified as a new member of the stem cell family due to the presence of fundamental stem cell properties (Bi et al., 2007). TDSCs are isolated through enzymatic digestion of tendon samples using type I collagenase, followed by filtering the resulting suspension through a 70 μm cell strainer (Baumgarten et al., 2025). The isolated cells are cultured at low density, and the isolated TDSCs are further identified by stem cell characterization assays (Wu et al., 2024; Chen et al., 2025; Zhang et al., 2011; Tan et al., 2012). Approximately 1%–2% of isolated TDSCs express stemness while simultaneously expressing tendon and cartilage-specific markers (Rui et al., 2010). TDSCs possess unique phenotypic characteristics. TDSCs, after undergoing continuous passages *in vitro*, often transform phenotypically and become less proliferative (Wu et al., 2024). However, TDSCs’ proliferation capacity and maintenance often require the addition of special media that mimic the natural tendon microenvironment. However, the culture systems used to maintain TDSCs vary significantly across different research groups. Commonly used media for TDSC culture include DMEM with low glucose DMEM supplemented with fetal bovine serum (FBS) and growth factors (Rui et al., 2010), standard DMEM (Chen et al., 2025), and alpha-Minimum Essential Medium (α-MEM) (Bi et al., 2007). In general, the concentration of FBS used to culture TDSCs varies from 10% to 20% (Bi et al., 2007; Chen et al., 2025; Tan et al., 2012; Rui et al., 2010). In addition, several low-molecular-weight compounds are often added to the media. L-glutamine (Tan et al., 2012; Rui et al., 2010; Rui et al., 2013), a low-molecular-weight compound, plays a crucial role in promoting TDSC proliferation. However, no standardized protocol for TDSC culture exists at present. The methodologies used to culture TDSCs vary significantly based on the study requirements (Figure 2).

TDSCs are characterized by the presence of fundamental stem cell properties, including clonogenicity, multipotency, and self-renewal capacity (Bi et al., 2007). The clonogenic potential of TDSCs is strong, as evidenced by their capacity to produce colony-forming units (CFUs) *in vitro*, indicating their potential to proliferate from a single cell to a population of cells. TDSCs have a higher proliferative potential compared to mature tenocytes, whereby they can proliferate and retain their undifferentiated state, a critical characteristic in the context of rapidly replacing lost cells after injury. Multipotency is a defining characteristic of TDSCs, whereby these cells can differentiate into various cell types upon specific differentiation conditions, whereby tenogenic differentiation is the primary and most fundamental differentiation pathway of TDSCs. TDSCs are capable of forming tendon-like tissues following *in vitro* expansion and *in vivo* transplantation (Bi et al., 2007). The cells within the regenerated tissue express tendon-specific markers, which include Scleraxis (Scx), Mohawk (Mkx), Tenomodulin (Tnmd), and Collagen Type I (Col I). Additionally, TDSCs can differentiate into osteocytes, chondrocytes, and adipocytes under certain conditions (Tan et al., 2012; Zhang M. et al., 2024). TDSCs maintain tendon tissue homeostasis under normal conditions and initiate repair mechanisms in response to injury. However, TDSCs can trigger pathological tissue remodeling and contribute to the pathogenesis of tendon disorders via aberrant signaling pathways (Chen et al., 2025; Chen M. et al., 2024; Konar et al., 2024; Guo et al., 2025). The detailed analysis of the properties and functions of TDSCs not only helps in understanding the pathogenesis of tendon disorders but is critical for developing an effective treatment strategy to improve the clinical outcome of tendon disorder patients.

### 2.2 The role of TDSCs in the treatment of tendon disorders

TDSCs play a significant role in the repair of tendons through a variety of synergistic mechanisms, of which the most fundamental is differentiation and replacement of tissues. In the aftermath of a tendon injury, TDSCs, either exogenously or endogenously administered, are attracted to the injury site by the influence of inflammatory mediators and chemokines present in the injured area (Chen et al., 2025; Konar et al., 2024). At the injury site, certain mechanical stimuli are responsible for the differentiation of TDSCs into the tenocyte lineage (He W. et al., 2024; Zhang et al., 2019; Dang et al., 2025). Physical interventions are often required in the treatment of tendons. Research has demonstrated that piezoelectric effects induced by ultrasound significantly enhance the proliferation of TDSCs (Luo et al., 2025). The differentiated tenocytes then go on to produce and secrete vital components of the extracellular matrix, which include Type I collagen, Type III collagen, elastin, and tenomodulin (Zhang Z. et al., 2021). These components finally form the structural framework of the tendon extracellular matrix, replacing scar tissue to form functional neo-tendon tissue. It is therefore vital to promote the process of tenogenic differentiation of TDSCs for them to function effectively. For instance, a bio-mimetic approach termed the “tendon patch” has been shown to induce tenogenic differentiation of TDSCs by inhibiting the AGEs/NF-κB/COX-2 pathway, thus reducing local tendon inflammation (Zhang T. et al., 2026). Additionally, the bioactive flavonoid icariin (ICA) has been shown to induce significant tenogenic differentiation of TDSCs and improve the ultrastructural organization of tendons (He et al., 2025). TDSCs possess significant promise as seed cells for tendon tissue engineering (Zhang S. et al., 2023). A recent set of studies based on the use of biomaterials has shown that chitosan hydrogel encapsulation of TDSCs (Wen et al., 2024) and a DNA hydrogel delivery system (TDSCs–Gel) (Ge et al., 2023). effectively promote tendon healing. Moreover, the targeted knockdown of CD248 by siRNA-loaded liposomes has been shown to improve the healing capacity of TDSCs (Wang Y. et al., 2023). While the findings of these studies point to significant promise for TDSCs in tissue repair, the majority of the studies are based on small animal models and lack double-blind studies.

In addition to their differentiation potential into new tenocytes, TDSCs are considered the main coordinator of the process of tendon injury repair. TDSCs significantly regulate inflammation and the immune environment, creating favorable conditions for tissue healing. Their paracrine effects are thought to impact tissue repair more than their differentiation capacity. Research confirms that TDSCs manage inflammatory responses via JNK and STAT3 pathways by secreting anti-inflammatory cytokines, thus balancing pro- and anti-inflammatory signals (Tarafder et al., 2017). This regulatory role is of great importance in the repair of tendons. In another study, it was found that TDSCs play an essential role in tendon repair by upregulating the expression of Col I and tenomodulin (TNMD), thus enhancing the reconstruction of the inflammatory environment and protecting tenocytes, thereby supporting tendon repair (Wang S. et al., 2023). Furthermore, TDSCs secrete small extracellular vesicles (sEVs) containing bioactive molecules under hypoxic conditions. The secretory vesicles have been found to significantly reduce inflammation and fibrosis, and increase the expression of tendon markers, thus improving the architecture and function of the tendon in a rat model of Achilles tendon injury (Wu et al., 2025). Genetically engineered TDSCs overexpressing hepatocyte growth factor (HGF) can enhance the healing of tendons by inhibiting the TGF-β1 signaling pathway, which reduces inflammation and fibrosis (ZHANG M. et al., 2021). TDSCs can also interact with immunocytes and maintain the balance of the immune response. TDSCs can inhibit the overactivation of T cells through the CD39/CD73-mediated adenosine pathway, which maintains the balance of the immune response and reduces immunocyte-mediated tissue injury (Altaie et al., 2025).

## 3 Application of exosomes in tendon-related disorders

Exosomes are membrane vesicles that are secreted by the cell and are nanosized, with diameters ranging from 30 to 200 nm (Sisia et al., 2026). Exosomes are membrane vesicles that contain bioactive molecules, which are embedded inside the membrane that is derived from the cell membrane (Kalluri and Mcandrews, 2023). Exosomal membranes are similar to lipid rafts, which are rich in cholesterol and sphingomyelin, providing the membrane with considerable structural stability (Rezaie et al., 2022). Exosomal proteins and nucleic acids are the main players that participate in the regulatory roles that exosomes play. Exosomes exhibit unique biological properties, which include stable physicochemical properties, intrinsic biocompatibility, and low immunogenicity (Zou et al., 2025). The lipid membrane of the exosomes acts to protect the encapsulated cargo from degradation by extracellular enzymes. It thus enables the stable, long-range transmission of molecular information. Due to the endogenous nature of the exosomes, as well as the membrane composition derived from the host cells, they tend to be highly biocompatible with low immunogenicity (Zou et al., 2025)., This greatly minimizes the chance of immune rejection. Exosomes thus offer a lot of promise as a natural drug delivery vehicle for therapeutic applications (Wang et al., 2025). The adhesion molecules present on the surface of the exosomes allow for targeted binding to the target cells (Kostyusheva et al., 2025). This makes the exosomes a tool for precise drug delivery (Kooijmans et al., 2021; Yang et al., 2026; Mehdizadeh et al., 2025). This specificity of delivery allows exosomes to transport therapeutic agents to areas that would be hard to target with traditional drugs. The variety and quantity of molecular cargo carried by exosomes depend on the type of cell that secretes them (D’angelo et al., 2025; Ljungström and Oltra, 2025). This inherent variability is responsible for the wide range of exosome function. Exosomes have been established as essential mediators of physiological processes, including intercellular communication, cell migration, and anti-tumor immune responses (He et al., 2026). A large number of studies have emphasized the prominent role of exosomes in intercellular communication, including the ability to modulate the function of the target cell (Wessler and Meisner-Kober, 2025). The exosomes carry out intercellular communication with target cells by means of interactions between ligands and receptors, membrane fusion, and the endocytosis of the membrane-bound vesicle (Wang et al., 2025; Xiang et al., 2024), thereby allowing local and systemic communication (Rezaie et al., 2022; Belényesi et al., 2025). Through this mechanism of intercellular communication, exosomes play an essential role in the development of different diseases. In oncology, it has been established that small molecule inhibitors of exosome secretion have therapeutic potential as an approach to slow the progression of cancer (Lee et al., 2024). On the other hand, exosomes have been widely utilized as an effective drug delivery system for cancer therapy, including bone (Zhang X. et al., 2026), gastrointestinal malignancies (Song et al., 2025), and central nervous system tumors (Xu et al., 2026).

Currently, most general exosomes investigated in tendinopathy treatment research are derived from non-tendon-resident cells. In terms of regulating tenocyte function, exosomes from young stem cells can improve the function of aged TDSCs and enhance their self-renewal and proliferative capacity by inhibiting the NF-κB pathway and upregulating SIRT1 expression (Han et al., 2024). Platelet-derived exosomes not only ameliorate senescence and ferroptosis in TDSCs, but also serve as a delivery vehicle for recombinant Yap1, inhibiting the senescence-associated phenotype of TDSCs by blocking ROS-mediated activation of the NF-κB signaling pathway (Chen D. et al., 2024; Lu et al., 2023). Adipose-derived stem cell exosomes upregulate the expression of tendon repair-related genes (e.g., RUNX2, Sox-9) in TDSCs (Fu et al., 2021), and exert therapeutic effects in acute tendon injury via the let-7c-5p/CRCT1/JAK2/STAT3 signaling pathway (Liu et al., 2025; Xu et al., 2019). Schwann cell-derived exosomes promote tenocyte migration and proliferation through the PTEN/PI3K/Akt signaling pathway (Chunbo et al., 2025). Bone marrow mesenchymal stem cell-derived exosomes facilitate TDSC proliferation and migration via paracrine signaling effects (Yu et al., 2020). Additionally, tenocyte-derived exosomes can induce tenogenic differentiation of mesenchymal stem cells in a TGF-β-dependent manner. Although all these general exosomes can participate in tendon injury repair to a certain extent, they exhibit significant differences compared with exosomes secreted by tendon-derived stem cells (TDSCs). Most general exosomes are derived from non-tendon tissue cells, lacking tendon tissue specificity. Their cargo composition has low compatibility with tendon physiological function and injury repair, resulting in limited targeting and repair efficiency. In contrast, TDSC-derived exosomes have unique advantages in the treatment of tendinopathy, which is closely related to the tissue specificity of their parent cells (Von Stade et al., 2025). As resident stem cells of tendon tissue, TDSCs secrete exosomes whose cargo composition is highly consistent with tendon physiological function and injury repair, endowing them with more precise regulatory capabilities and superior repair effects that are unmatched by other non-tendon-derived exosomes (Lin et al., 2025).

## 4 Research on exosomes derived from TDSCs

### 4.1 Isolation and identification of exosomes derived from TDSCs

Ultracentrifugation continues to be a core method in the isolation of exosomes. However, the use of a variety of methods has been found to produce highly purified exosomes, as well as preserve the structural integrity of the exosomes. For example, the integration of tangential flow filtration (TFF) with size exclusion chromatography (SEC) represents an efficient approach that maintains structural integrity. In this method, tangential flow filtration is used to rapidly concentrate large volumes of conditioned media, followed by SEC to produce highly purified exosomes (Gurriaran-Rodriguez et al., 2024). In other studies, a combination of differential centrifugation and SEC has been found to be a potent method in the isolation of exosomes.

Subsequent to this, an extensive analysis of the structure and function of exosomes is necessary. The structure of exosomes is usually studied by transmission electron microscopy, wherein classical exosomes are identified by their cup-shaped or biconcave structures with a lipid bilayer membrane (Kalluri and Mcandrews, 2023). The size distribution and concentration of exosomes are usually measured by nanoparticle tracking analysis, wherein exosomes isolated from TDSCs range from 30 to 150 nm (Xue et al., 2022). The presence of classical positive markers, which include transmembrane proteins, is studied by performing a Western blot assay (Xue et al., 2022; Liu et al., 2024; Li et al., 2024). The function of exosomes is critical and is usually studied while performing tendon regeneration research. CCK-8 assay, scratch wound assay, and transwell migration assay are some of the assays that are used to assess the function of exosomes on TDSCs/tenocytes (Li et al., 2021). Evaluation of tenogenic differentiation involves co-culturing exosomes with TDSCs and examining the upregulation of tendon-specific differentiation markers. To elucidate the underlying molecular mechanisms, techniques such as Western blotting and quantitative real-time PCR (qRT-PCR) are applied to analyze signaling pathways modulated by exosomes. Moreover, optical biosensors have emerged as advanced analytical tools for exosome detection and characterization. By integrating nanomaterials and engineered recognition elements, these biosensors significantly enhance detection sensitivity and specificity, demonstrating considerable potential in exosome research (Vafadar et al., 2026).

### 4.2 Biological functions of TDSC-derived exosomes

Exosomes derived from tendon-derived stem cells (TDSCs) can promote the proliferation and collagen synthesis of tenocytes and TDSCs via the synergistic regulation of multiple mRNAs and signaling pathways. The encapsulated mRNAs of COL1A1 and COL3A1 directly participate in collagen synthesis and assembly, thereby upregulating the expression of COL1 and COL3 in tenocytes. Meanwhile, tendon-specific mRNAs such as SCX and TNMD significantly enhance the expression of tendon-specific biomarkers and drive tenocyte differentiation toward a mature phenotype. A study published in 2022 confirmed that TDSC-derived exosomes facilitate tenocyte differentiation and migration. Mechanistically, highly expressed VEGFA mRNA within these exosomes positively regulates the proliferation and metabolism of TDSCs by activating the mTOR signaling pathway, maintaining tendon tissue homeostasis. In contrast, aberrant VEGFA expression disrupts TDSC proliferation and impairs tendon repair (Xue et al., 2022). Furthermore, TDSC-derived exosomes enhance tenocyte proliferation, migration, COL1 production, and the expression of tendon-specific markers (Song et al., 2022). During this process, encapsulated CXCL12 mRNA establishes an ordered cell migration gradient, guiding the targeted homing of tenocytes and TDSCs toward injured sites to provide sufficient cellular resources for tissue repair (Xue et al., 2022). Building on these findings, increasing studies have further explored the detailed mechanisms underlying TDSC-derived exosomes.

TDSC-derived exosomes also play a vital role in balancing the extracellular matrix of tenocytes (Wang et al., 2019). Encapsulated MMP-3 and TIMP-1 mRNAs modulate the balance between matrix metalloproteinases and their inhibitors, restraining excessive degradation of the extracellular matrix and preventing abnormal fibrosis during tendon healing. Notably, TDSC-derived exosomes are rich in transforming growth factor-β (TGF-β), which activates the TGF-β/Smad3 signaling pathway to robustly promote TDSC proliferation and migration, accelerate the synthesis of tendon matrix proteins, and lay a molecular foundation for the tenogenic differentiation of TDSCs (Li et al., 2021). In addition, abundant anti-aging signals in TDSC-derived exosomes alleviate the senescent phenotype of aged TDSCs, restore their reparative capacity, and preserve their tenogenic potential (Jin et al., 2023). This protective function is mainly mediated by encapsulated SIRT1 mRNA, which inhibits the NF-κB signaling pathway to reduce the expression of senescence-related genes and delay TDSC senescence. Moreover, SIRT1 cooperates with miR-337-3p to suppress Caspase3 expression, attenuate tenocyte apoptosis, and further strengthen the reparative effects (An et al., 2024).

In addition, TDSC-derived exosomes exert anti-inflammatory and antioxidant effects. Preclinical studies have demonstrated that these exosomes markedly inhibit inflammation and cellular apoptosis, enabling high-quality healing of damaged tendons. The core mechanism involves encapsulated IL-10 and TGF-β1 mRNAs, which downregulate pro-inflammatory factors such as TNF-α and IL-6, while activating the PI3K-Akt signaling pathway to suppress tenocyte apoptosis (Zhang et al., 2020). Another study revealed that TDSC-derived exosomes protect tenocytes against oxidative stress and serum deprivation (Song et al., 2022). Specifically, the enriched miR-144-3p targets ARID1A to enhance tenocyte proliferation and migration. A latest study reported in January 2025 combined TDSC-derived exosomes with hydrogels. This composite system promoted the polarization of macrophages from the pro-inflammatory M1 phenotype to the anti-inflammatory M2 phenotype via the PI3K-Akt and MAPK signaling pathways, while facilitating tissue regeneration and ordered collagen deposition (Li et al., 2025), further clarifying the anti-inflammatory and pro-collagen effects of TDSC-derived exosomes. In summary, TDSC-derived exosomes exert therapeutic effects through a core pattern of multi-molecular coordination and multi-pathway crosstalk. By carrying functional mRNAs (COL1A1, COL3A1, SCX, TNMD, VEGFA, CXCL12, SIRT1, IL-10, TGF-β1) and non-coding RNAs (miR-337-3p, miR-144-3p), these exosomes activate pro-repair signaling cascades including mTOR, TGF-β/Smad3, PI3K-Akt and MAPK, while suppressing the pro-inflammatory and pro-senescent NF-κB pathway. They ultimately form a comprehensive regulatory network covering proliferation and differentiation, anti-inflammation and antioxidation, anti-senescence, and extracellular matrix homeostasis, thereby participating in the entire process of tendon injury repair (Maleki et al., 2026).

### 4.3 Application potential of TDSC-derived exosomes

TDSC-Exos have demonstrated significant therapeutic potential in tendon regeneration, including conditions such as tendon injuries, tendinopathy, and enthesis injuries, due to their robust anti-inflammatory and regenerative properties (Zhang et al., 2020; He Y. et al., 2024; Zhang et al., 2024b; Zhang et al., 2024c). Exosomes derived from TDSCs also show promise for drug delivery and gene therapy, which can be attributed to the following properties: low toxicity, minimal immunogenicity, high uptake, high biocompatibility, and stability (Langellotto et al., 2025). Two studies have investigated TDSC-Exos as an approach to gene therapy. The studies found that TDSC-Exos can accelerate the healing of injured Achilles tendons by regulating miR-145-3p (An et al., 2024). The second study found that exosomes of TDSCs that overexpress miR-337-3p can protect tenocytes from apoptosis by regulating CASP3 (Zhang T. et al., 2023). All these gene therapy approaches have provided promising directions for the treatment of tendon disorders and improving patient outcomes. Recently, cell-free therapeutic strategies, especially exosome therapy, have been of great interest. Among these strategies, TDSC-derived exosomes have emerged as particularly promising tools for promoting high-quality tendon repair due to their tissue-specificity, potent regenerative capacities, and minimal immunogenicity. Their therapeutic potential is highlighted through several key mechanisms.

#### 4.3.1 Promoting endogenous tendon regeneration and repair

The main therapeutic value of TDSCs-Exos is their ability to activate and regulate endogenous repair mechanisms. TDSCs-Exos, being natural mediators of intercellular communication, play a significant role in the modulation of local repair responses by directly delivering a variety of bioactive signals to the target cells in the area of injury. A significant volume of studies has demonstrated that TDSCs-Exos significantly stimulate the proliferation and migration of TDSCs and tenocytes through autocrine and paracrine effects, thus offering a cellular basis for efficient tendon regeneration. Specifically, TDSC-Exos containing TGF-β can stimulate the proliferation and migration of TDSCs through the activation of the TGF-β-Smad2/3 and ERK1/2 signaling pathways (Li et al., 2021). TDSC-Exos, meanwhile, have shown potency in the upregulation of tendon-specific extracellular matrix (ECM) molecules, like type I collagen and tenomodulin, while simultaneously inhibiting the activity of matrix metalloproteinase-3 (MMP-3). The aforementioned process maintains a balance between the synthesis and degradation of the ECM, hence encouraging the regeneration of functional and structurally sound tendons that do not form scars (Wang et al., 2019). The therapeutic functions of TDSC-Exos are closely linked to their encapsulated microRNAs (miRNAs). For instance, TDSC-Exos promote tenocyte proliferation and migration by transferring miR-144-3p, which specifically targets and inhibits ARID1A expression (Song et al., 2022). Zhang et al. demonstrated that TDSC-derived small extracellular vesicles (TDSC-sEVs) enhance tendon repair through miR-145-3p regulation (Zhang T. et al., 2023), while research by An et al. found that miR-337-3p-overexpressing TDSC-Exos effectively inhibit tenocyte apoptosis via CASP3 targeting, providing a novel approach for cellular protection at injury sites (An et al., 2024). Collectively, the findings from the aforementioned studies indicate that the therapeutic potential of exosomes can be improved by the enrichment of particular miRNAs via engineered approaches. However, it has been noticed that the majority of the current studies are based on *in vitro* studies and small animal models, which might not accurately represent the human physiological conditions, considering the complexity of the signaling pathways and therapeutic potential. In addition, the diversity in the source of the TDSCs, culture conditions, and exosome isolation procedures might influence the therapeutic potential of the bioactive content of the exosomes. In addition, the therapeutic potential of the individual miRNAs might be overlooked, which might influence the therapeutic outcome or result in off-target effects.

#### 4.3.2 Regulating the immune microenvironment and breaking the senescence-associated vicious cycle

Tendon healing largely depends on the immune-inflammatory response following a tendon injury. While a moderate immune-inflammatory response is essential for healing, a severe immune-inflammatory response might result in poor healing and fibrosis. TDSC-Exos exhibit significant immunomodulatory potential, particularly in the treatment of chronic and aging-related refractory tendon injuries. The main role of TDSC-Exos is that they induce a shift from the immune-inflammatory M1 macrophages to the anti-inflammatory and pro-reparative M2 macrophages. He et al. showed that TDSCs, which are enriched with miR-21a-5p, regulate the PDCD4/AKT/mTOR signaling pathway, which inhibits M1 macrophage polarization and induces M2 macrophage polarization. This modulation markedly improved the inflammatory microenvironment and enhanced tendon-bone interface healing in a rotator cuff repair model (He Y. et al., 2024). Such immunoregulatory effects are essential for controlling early inflammation and facilitating the transition to the regenerative phase. In aged individuals or chronic tendon injuries, a detrimental feedback loop may develop between senescent TDSCs and immune cells, hindering tissue repair. Conditioned medium derived from senescent TDSCs (s-TDSCs) promotes macrophage polarization toward the M1 phenotype, whereas conditioned medium from M1 macrophages further accelerates TDSC senescence, forming a “positive feedback loop.” Exosomes derived from healthy, young TDSCs (h-TDSC-Exos) can disrupt this cycle by inducing macrophage polarization toward the M2 phenotype, thereby mitigating the senescent phenotype of s-TDSCs and enhancing tendon-bone healing in aged chronic rotator cuff injury models (Zhang et al., 2024b). These findings show that h-TDSC-Exos not just enhance regeneration but also influence the tissue microenvironment. Immune modulation is associated with certain risks too. During an injury scenario where the chance of infection cannot be completely excluded, an overzealous suppression of M1 macrophage activity might lead to poor outcomes by compromising the ability to control pathogens. It is also noteworthy that the tissue immune environment in chronic tendinopathy is extremely complex, with macrophage phenotypes extending beyond the M1/M2 spectrum. The regulatory activity of TDSC-Exos might not influence the entire spectrum of this complex immune environment. Finally, the long-term safety of injecting exosomes from young healthy donors into old/diseased tissues and the effect on natural aging processes need to be evaluated extensively.

#### 4.3.3 Serving as a carrier for advanced drug delivery and tissue engineering platforms

In addition to the inherent therapeutic capabilities of exosomes, those derived from TDSC-Exos offer the best platform for a drug delivery system at the nanoscale. These exosomes can be incorporated into biomaterials to create the best tissue-engineered scaffold. Although the direct administration of exosomes alone has limited retention time within the body, which allows the exosomes to diffuse quickly, the incorporation of exosomes into biomaterials allows for the controlled release of the exosomes. For example, Cui et al. developed a biofunctionalized scaffold using collagen-targeted exosomes derived from TDSCs (TDSC-EVs) to create a decellularized tendon scaffold. This biofunctionalized scaffold significantly improved tendon regeneration *in vitro* and *in vivo* (Cui et al., 2023). Furthermore, using 3D bioprinting technology, scaffolds loaded with TDSC-Exos have been designed to repair massive irreparable rotator cuff tears. These scaffolds not only provide mechanical support but also facilitate sustained exosome release, promoting tenogenic differentiation with therapeutic outcomes comparable to autologous fascia grafting (Zhang et al., 2024c). Another approach involved encapsulating TDSC-Exos within hydrogels derived from decellularized tendon matrices; this combination demonstrated synergistic effects, effectively enhancing tendon regeneration (Li et al., 2025). Additionally, TDSCs can be preconditioned or genetically engineered to generate “engineered exosomes” with improved functionality. For instance, hypoxic culture conditions for TDSCs yield small extracellular vesicles (sEVs) enriched with bioactive molecules, which more effectively promote tendon healing via the HIF-1α/miR-145-3p pathway (Wu et al., 2025). Despite these encouraging developments, the complexity and high costs involved in advanced engineering techniques, along with a lack of standardized guidelines for production, remain major challenges for the production of exosomes. The challenges include the efficiency of drug loading, control over exosome release, and targeting efficiency.

#### 4.3.4 Prospects for scalable production and clinical translation

**FIGURE 3:**
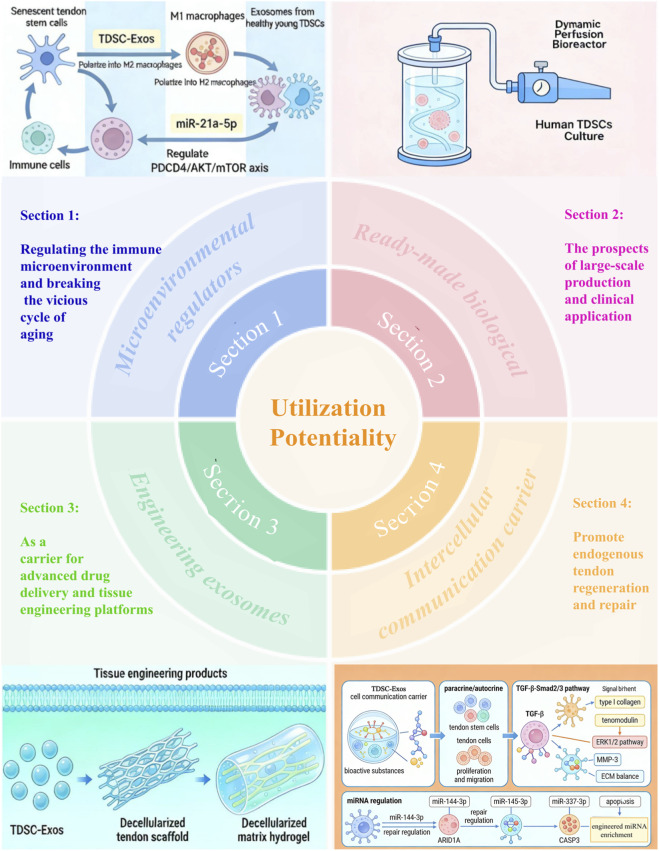
Application Potential of TDSC-Exos in Tendon Diseases. TDSC-Exos exert versatile therapeutic effects across four core dimensions: (1) as microenvironmental regulators, they reverse the senescent vicious cycle by polarizing M1 macrophages to M2 via the miR-21a-5p/PDCD4/AKT/mTOR axis; (2) as ready-made biological agents, they enable scalable clinical translation through large-scale culture in dynamic perfusion bioreactors; (3) as engineered exosomes, they serve as advanced drug delivery vehicles integrated into tissue engineering scaffolds (e.g., decellularized tendon scaffolds, hydrogels); (4) as intercellular communication carriers, they promote endogenous tendon regeneration by delivering bioactive molecules (miRNAs, growth factors) to activate pro-repair signaling pathways (TGF-β/Smad2/3, ERK1/2) and regulate extracellular matrix homeostasis.

Scalable and standardized production of TDSC-derived exosomes is essential to integrate them clinically. Studies by Clerici et al. have indicated that human TDSCs cultured in dynamic perfusion bioreactor systems produce three times more exosome-derived protein compared to traditional static cultures (Clerici et al., 2025). Such effective production strategies of TDSC-derived exosomes have provided significant implications to fulfill the high demand for TDSC-Exos. When combined with advanced storage strategies, TDSC-Exos have immense potential to be developed as off-the-shelf biological therapeutics (Figure 3).

## 5 Summary and prospects

### 5.1 Advantages of exosome-mediated TDSCs therapeutic strategies

In recent years, stem cell–based tissue engineering strategies have demonstrated considerable therapeutic potential. However, the clinical application of cell-based therapies has been constrained by concerns regarding immune rejection (Hovatta et al., 2011), tumorigenicity (Yamaguchi et al., 2024; Jiang et al., 2023), and ethical issues (Harris et al., 2022). In this context, exosome-mediated TDSC therapy has emerged as a prominent frontier in regenerative medicine. By utilizing exosomes secreted by TDSCs to exert a “cell-free therapeutic effect,” (Chen et al., 2026) this strategy offers a novel approach to tendon repair that combines high efficacy with enhanced safety, thereby promoting a potential paradigm shift in treatment strategies. The exosome-based TDSC strategy changes the paradigm of cell-based therapy from cell transplantation to bioactive cargo utilization. Through the exploitation of the intrinsic advantages of exosomes, which include low immunogenicity, the absence of tumorigenic risk, high stability, and dosage control (Sgaglione et al., 2025; Ren, 2019), this therapeutic modality exploits the intrinsic healing capacity of TDSCs in a safe, less invasive manner with fewer side effects. As such, this modality holds tremendous promise for the treatment of tendon pathology. In addition, the intrinsic versatility of the exosome-based modality makes it applicable to a wide range of tendon disorders, including bioengineering and drug delivery systems (Serrano et al., 2025).

### 5.2 Challenges

Despite its promising potential, the exosome-mediated TDSC therapeutic strategy faces two major challenges: scalable production and stringent quality control (Verma and Arora, 2025). These obstacles largely stem from the absence of standardized protocols for exosome isolation and purification (Meng et al., 2020). Current extraction and characterization techniques are technically demanding and time-consuming, highlighting the need for more efficient and scalable purification technologies capable of producing high-yield, high-purity exosomes. Additionally, clinical trials evaluating exosome-based therapies remain limited, partly due to suboptimal drug-loading efficiency and insufficient production capacity for clinical-grade manufacturing (Meng et al., 2020). Meanwhile, the short half-life of exosomes *in vivo* highlights the need to explore their long-term therapeutic effects further. The eventual clinical application of exosome therapies is expected to follow a stepwise approach. However, the outcomes of exosome therapy remain uncertain, possibly due to the complex molecular pathways through which exosomes influence disease processes. Limited understanding of the exact mechanisms of exosome action hampers their effective clinical application. Future studies should focus on improvements in exosome preparation, characterization, and optimization methods, including the latest progressive methods of exosome extraction, such as extrusion (Zhou et al., 2026), determination of the optimal dosage of exosomes for treatment, and evaluation of the mechanisms and treatment potential of exosomes in various diseases. In addition, the adverse effects of exosome treatment must be examined. Exosomes may contain proteins, nucleic acids, or other substances that may lead to adverse effects in humans, including cancer development, even in non-oncological treatment regimens. Therefore, the treatment potential of exosomes must be carefully examined. The inclusion of any new treatment modality in clinical practice follows a stepwise approach. As a carrier in precision medicine, exosomes have the potential to be genetically, chemically, and nanomaterially modified for the development of novel targeted delivery systems. Improved understanding of exosome isolation, modification, and use can significantly enhance their therapeutic research value. Targeted exosomes with improved targeting abilities show promise in treating various diseases. To overcome the problem of rapid clearance of exosomes from the body, researchers have explored the use of advanced biomaterial delivery systems, including injectable hydrogels (Song et al., 2023; Astaneh et al., 2026; Xu Ht. et al., 2025; Xu et al., 2025b) or decellularized tendon scaffolds (Cui et al., 2023). These novel exosome-based carrier and scaffold systems enable localized sustained release at injury sites, substantially enhancing exosome retention and therapeutic efficacy (Villani et al., 2024; Xu et al., 2025c). Such strategies not only offer revolutionary approaches for managing difficult-to-treat tendon injuries but also represent significant future directions in regenerative medicine toward “cell-free therapy” and “standardized biologics.” Ultimately, exosomes hold great promise as effective clinical intervention tools for disease treatment.

## References

[B1] Altaie A. Simone D. Mcdermott N. Owston H. Attar M. Jin L. (2025). Healthy human enthesis stromal cells mediate immunoregulation *via* the CD39/CD73 adenosine ectonucleotidase pathway. Ann. Rheum. Dis. 84 (12), 1995–2007. 10.1016/j.ard.2025.09.001 41058388

[B2] An Q. Zhou Z. Xu C. Xiao Q. (2024). Exosomes derived from mir-337-3p over-expressing tendon stem cells protect against apoptosis of tenocytes *via* targeting caspase3. BMC Musculoskelet. Disord. 25 (1), 561. 10.1186/s12891-024-07691-9 39030590 PMC11264700

[B3] Astaneh M. E. Baldaniya L. Singh A. Hussen A. Hashemzadeh A. Fereydouni N. (2026). Exosome-enhanced injectable hydrogels: a comprehensive review on their emerging role in wound healing. Health Sci. Rep. 9 (1), e71694. 10.1002/hsr2.71694 41477216 PMC12748068

[B4] Baumgarten K. M. Sandhurst E. S. Sun H. (2025). Can human growth hormone accelerate tendon and ligament injury recovery? Sports Health 17 (2), 299–304. 10.1177/19417381241245938 38618948 PMC11571130

[B5] BeléNYESI S. K. Patmore S. O'Driscoll L. (2025). Extracellular vesicles and the tumour microenvironment. Biochim. Biophys. Acta Rev. Cancer 1880 (2), 189275. 39900204 10.1016/j.bbcan.2025.189275

[B6] Bi Y. Ehirchiou D. Kilts T. M. Inkson C. A. Embree M. C. Sonoyama W. (2007). Identification of tendon stem/progenitor cells and the role of the extracellular matrix in their niche. Nat. Med. 13 (10), 1219–1227. 10.1038/nm1630 17828274

[B7] Chen M. Zou F. Wang P. Hu W. Shen P. Wu X. (2024a). Dual-barb microneedle with JAK/STAT inhibitor-loaded nanovesicles encapsulation for tendinopathy. Adv. Healthc. Mater 13 (28), e2401512. 10.1002/adhm.202401512 39030889

[B8] Chen D. Tang Q. Song W. He Y. (2024b). Platelet-derived exosomes alleviate tendon stem/progenitor cell senescence and ferroptosis by regulating AMPK/Nrf2/GPX4 signaling and improve tendon-bone junction regeneration in rats. J. Orthop. Surg. Res. 19 (1), 382. 10.1186/s13018-024-04869-8 38943181 PMC11212425

[B9] Chen S. Lin Y. Yang H. Li Z. Li S. Chen D. (2025). A CD26(+) tendon stem progenitor cell population contributes to tendon repair and heterotopic ossification. Nat. Commun. 16 (1), 749. 10.1038/s41467-025-56112-5 39820504 PMC11739514

[B10] Chen H. Li N. Cai Y. Ma C. Ye Y. Shi X. (2026). Exosomes in neurodegenerative diseases: therapeutic potential and modification methods. Neural Regen. Res. 21 (2), 478–490. 10.4103/NRR.NRR-D-24-00720 40326981 PMC12220696

[B11] Chunbo L. Jia L. Jian Z. Yaxian G. Chenwei G. Cong X. (2025). Exosomes derived from schwann cells (SCDE) promote tendon repair through the PTEN/PI3K/Akt signaling pathway. J. Orthop. Surg. Res. 20 (1), 911. 10.1186/s13018-025-06352-4 41121340 PMC12539145

[B12] Clerici M. Ciardulli M. C. Lamparelli E. P. Lovecchio J. Giordano E. Dale T. P. (2025). Human tendon stem/progenitor cell-derived extracellular vesicle production promoted by dynamic culture. Artif. Cells Nanomed Biotechnol. 53 (1), 1–16. 10.1080/21691401.2025.2475099 40063517

[B13] Cui J. Zhang Y. J. Li X. Luo J. J. Zhao L. L. Xie X. Y. (2023). Decellularized tendon scaffolds loaded with collagen targeted extracellular vesicles from tendon-derived stem cells facilitate tendon regeneration. J. Control Release 360, 842–857. 10.1016/j.jconrel.2023.07.032 37478916

[B14] D'Angelo G. Stahl P. D. Raposo G. (2025). The cell biology of extracellular vesicles: a jigsaw puzzle with a myriad of pieces. Curr. Opin. Cell Biol. 94, 102519. 10.1016/j.ceb.2025.102519 40267602

[B15] Dang J. Zhang Z. Fu J. Sun L. Shi Y. Wang L. (2025). Regulating inflammation microenvironment and tenogenic differentiation as sequential therapy promotes tendon healing in diabetic rats. J. Orthop. Transl. 53, 63–81. 10.1016/j.jot.2025.04.015 PMC12173140 40529900

[B16] Deng J. Oosterhof J. J. Eygendaal D. Breda S. J. Oei E. H. G. de Vos R. J. (2025). Long-term prognosis of athletes with patellar tendinopathy receiving physical therapy: patient-reported outcomes at 5-Year Follow-up. Am. J. Sports Med. 53 (7), 1568–1576. 10.1177/03635465251336466 40356204 PMC12125489

[B17] Fu G. Lu L. Pan Z. Fan A. Yin F. (2021). Adipose-derived stem cell exosomes facilitate rotator cuff repair by mediating tendon-derived stem cells. Regen. Med. 16 (4), 359–372. 10.2217/rme-2021-0004 33871287

[B18] Ge Z. Li W. Zhao R. Xiong W. Wang D. Tang Y. (2023). Programmable DNA hydrogel provides suitable microenvironment for enhancing TSPCS therapy in healing of tendinopathy. Small 19 (32), e2207231. 10.1002/smll.202207231 37066733

[B19] GBD 2021 Other Musculoskeletal Disorders Collaborators (2023). Global, regional, and national burden of other musculoskeletal disorders, 1990-2020, and projections to 2050: a systematic analysis of the global burden of disease study 2021. Lancet Rheumatol. 5(11): e670-e682. 10.1016/S2665-9913(23)00232-1 37927903 PMC10620749

[B20] Guo H. Cao H. Lu Q. Gu Z. Feng G. (2025). TNF-α induces premature senescence in tendon stem cells *via* the NF-κB and p53/p21/cyclin E/CDK2 signaling pathways. Int. J. Mol. Med. 56 (3), 140. 10.3892/ijmm.2025.5581 40641112 PMC12270418

[B21] Gurriaran-Rodriguez U. De Repentigny Y. Kothary R. Rudnicki M. A. (2024). Isolation of small extracellular vesicles from regenerating muscle tissue using tangential flow filtration and size exclusion chromatography. Skelet. Muscle 14 (1), 22. 10.1186/s13395-024-00355-1 39394606 PMC11468478

[B22] Han W. Gu D. Li X. Chen H. Tao X. Chen L. (2024). Young TSPC-derived exosomal circPVT1 ameliorates aging-impaired cell function *via* SIRT1/NF-κB. Tissue Eng. Part C Methods 30 (6), 248–254. 10.1089/ten.tec.2024.0057 38842177

[B23] Harris A. R. Walker M. J. Gilbert F. (2022). Ethical and regulatory issues of stem cell-derived 3-dimensional organoid and tissue therapy for personalised regenerative medicine. BMC Med. 20 (1), 499. 10.1186/s12916-022-02710-9 36575403 PMC9795739

[B24] He W. Jiang C. Zhou P. Hu X. Gu X. Zhang S. (2024a). Role of tendon-derived stem cells in tendon and ligament repair: focus on tissue engineer. Front. Bioeng. Biotechnol. 12, 1357696. 10.3389/fbioe.2024.1357696 39175617 PMC11338810

[B25] He Y. Lu S. Chen W. Yang L. Li F. Zhou P. (2024b). Exosomes derived from tendon stem/progenitor cells enhance tendon-bone interface healing after rotator cuff repair in a rat model. Bioact. Mater 40, 484–502. 10.1016/j.bioactmat.2024.06.014 39040569 PMC11260958

[B26] He Z. Zeng S. Qin B. Liu G. Liu H. Bao D. (2025). Investigation on the role of icariin in tendon injury repair: focusing on tendon stem cell differentiation. J. Orthop. Surg. Res. 20 (1), 379. 10.1186/s13018-025-05784-2 40234966 PMC12001499

[B27] He T. Wang Y. Li X. Xiang C. Tan S. Fan R. (2026). Advancements in the study of exosomes in disease diagnosis and treatment. Int. J. Pharm. 689, 126471. 10.1016/j.ijpharm.2025.126471 41380924

[B28] Hefferan S. A. Blaker C. L. Ashton D. M. Little C. B. Clarke E. C. (2025). Structural variations of tendons: a systematic search and narrative review of histological differences between tendons, tendon regions, sex, and age. J. Orthop. Res. 43 (5), 994–1011. 10.1002/jor.26060 40012190 PMC11982604

[B29] Hovatta O. (2011). in Translational Stem Cell Research: Issues Beyond the Debate on the Moral Status of the Human Embryo. Editors HUG K. HERMERéN G. (Totowa, NJ: Humana Press), 103–110.The Obstacles on the Road to Clinical Applications of Stem Cell-based Therapies: What has been Done to Overcome These Obstacles and what Remains to be Done?

[B30] JäRVINEN T. A. Kannus P. Maffulli N. Khan K. M. (2005). Achilles tendon disorders: etiology and epidemiology. Foot Ankle Clin. 10 (2), 255–266. 10.1016/j.fcl.2005.01.013 15922917

[B31] Jiang L. Lu J. Chen Y. Lyu K. Long L. Wang X. (2023). Mesenchymal stem cells: an efficient cell therapy for tendon repair (review). Int. J. Mol. Med. 52 (2), 70. 10.3892/ijmm.2023.5273 37387410 PMC10373123

[B32] Jin S. Wang Y. Wu X. Li Z. Zhu L. Niu Y. (2023). Young exosome bio-nanoparticles restore aging-impaired tendon stem/progenitor cell function and reparative capacity. Adv. Mater 35 (18), e2211602. 10.1002/adma.202211602 36779444

[B33] Kalluri R. Mcandrews K. M. (2023). The role of extracellular vesicles in cancer. Cell 186 (8), 1610–1626. 10.1016/j.cell.2023.03.010 37059067 PMC10484374

[B34] Konar S. Leung S. Tay M. L. Coleman B. Dalbeth N. Cornish J. (2024). Novel *in vitro* platform for studying the cell response to healthy and diseased tendon matrices. ACS Biomater. Sci. Eng. 10 (5), 3293–3305. 10.1021/acsbiomaterials.4c00414 38666422

[B35] Kooijmans S. A. A. De Jong O. G. Schiffelers R. M. (2021). Exploring interactions between extracellular vesicles and cells for innovative drug delivery system design. Adv. Drug Deliv. Rev. 173, 252–278. 10.1016/j.addr.2021.03.017 33798644

[B36] Kostyusheva A. Romano E. Yan N. Lopus M. Zamyatnin A. A. Jr. Parodi A. (2025). Breaking barriers in targeted therapy: advancing exosome isolation, engineering, and imaging. Adv. Drug Deliv. Rev. 218, 115522. 10.1016/j.addr.2025.115522 39855273

[B37] Kwan K. Y. C. Ng K. W. K. Rao Y. Zhu C. Qi S. Tuan R. S. (2023). Effect of aging on tendon biology, biomechanics and implications for treatment approaches. Int. J. Mol. Sci. 24 (20), 15183. 10.3390/ijms242015183 37894875 PMC10607611

[B38] Langellotto M. D. Rassu G. Serri C. Demartis S. Giunchedi P. Gavini E. (2025). Plant-derived extracellular vesicles: a synergetic combination of a drug delivery system and a source of natural bioactive compounds. Drug Deliv. Transl. Res. 15 (3), 831–845. 10.1007/s13346-024-01698-4 39196501 PMC11782344

[B39] Lee Y. J. Shin K. J. Chae Y. C. (2024). Regulation of cargo selection in exosome biogenesis and its biomedical applications in cancer. Exp. Mol. Med. 56 (4), 877–889. 10.1038/s12276-024-01209-y 38580812 PMC11059157

[B40] Li M. Jia J. Li S. Cui B. Huang J. Guo Z. (2021). Exosomes derived from tendon stem cells promote cell proliferation and migration through the TGF β signal pathway. Biochem. Biophys. Res. Commun. 536, 88–94. 10.1016/j.bbrc.2020.12.057 33370718

[B41] Li M. Shi L. Chen X. Yi D. Ding Y. Chen J. (2024). *In-situ* gelation of fibrin gel encapsulating platelet-rich plasma-derived exosomes promotes rotator cuff healing. Commun. Biol. 7 (1), 205. 10.1038/s42003-024-05882-7 38374439 PMC10876555

[B42] Li D. Li S. He S. He H. Yuan G. Ma B. (2025). Restoring tendon microenvironment in tendinopathy: macrophage modulation and tendon regeneration with injectable tendon hydrogel and tendon-derived stem cells exosomes. Bioact. Mater 47, 152–169. 10.1016/j.bioactmat.2025.01.016 39906648 PMC11791013

[B43] Lin K. Hu X. Yan J. Gao R. Lin S. Zhou S. (2025). Exosomal miR-212-5p promotes tendon repair *via* targeting FOXO1 to activate PP1A/YAP1 signaling. Commun. Biol. 8 (1), 1798. 10.1038/s42003-025-09210-5 41326793 PMC12722425

[B44] Linsell L. Dawson J. Zondervan K. Rose P. Randall T. Fitzpatrick R. (2006). Prevalence and incidence of adults consulting for shoulder conditions in UK primary care; patterns of diagnosis and referral. Rheumatol. Oxf. 45 (2), 215–221. 10.1093/rheumatology/kei139 16263781

[B45] Liu X. Chen Y. Zhang T. (2024). Mechanism study of BMSC-Exosomes combined with hyaluronic acid gel in the treatment of posttraumatic osteoarthritis. Heliyon 10 (14), e34192. 10.1016/j.heliyon.2024.e34192 39100446 PMC11295849

[B46] Liu H. Zhang A. Shi M. Zhang J. Zhang T. Lu W. (2025). The treatment of acute tendon injury with small extracellular vesicles originating from TNFAIP6− ADSCs subpopoulation both *in vitro* and *in vivo* . Stem Cell Res. and Ther. 17 (1), 61. 10.1186/s13287-025-04789-2 41469712 PMC12866520

[B47] LjungströM M. Oltra E. (2025). Methods for extracellular vesicle isolation: relevance for encapsulated miRNAs in disease diagnosis and treatment. Genes (Basel) 16 (3), 330. 10.3390/genes16030330 40149481 PMC11942051

[B48] Lu J. Yang X. He C. Chen Y. Li C. Li S. (2023). Rejuvenation of tendon stem/progenitor cells for functional tendon regeneration through platelet-derived exosomes loaded with recombinant Yap1. Acta Biomater. 161, 80–99. 10.1016/j.actbio.2023.02.018 36804538

[B49] Luo R. Xiong Y. Li J. Xiao M. Bai Y. Xu Z. (2025). Piezoelectric injectable anti-adhesive hydrogel to promote endogenous healing of tendon injuries. Adv. Mater 37 (40), e2501306. 10.1002/adma.202501306 40658817

[B50] Maffulli N. Wong J. Almekinders L. C. (2003). Types and epidemiology of tendinopathy. Clin. Sports Med. 22 (4), 675–692. 10.1016/s0278-5919(03)00004-8 14560540

[B51] Maffulli N. Cuozzo F. Migliorini F. Oliva F. (2023). The tendon unit: biochemical, biomechanical, hormonal influences. J. Orthop. Surg. Res. 18 (1), 311. 10.1186/s13018-023-03796-4 37085854 PMC10120196

[B52] Maleki E. Karimizade A. Esfandiary F. Karimpour Malekshah A. Mirzaei M. Talebpour Amiri F. (2026). Regenerative effects of secretome from tendon-derived stem cells and treadmill training on achilles tendon healing in rats. Histochem Cell Biol. 164 (1), 11. 10.1007/s00418-026-02460-2 41779050

[B53] Mehdizadeh S. Mamaghani M. Hassanikia S. Pilehvar Y. Ertas Y. N. (2025). Exosome-powered neuropharmaceutics: unlocking the blood-brain barrier for next-gen therapies. J. Nanobiotechnology 23 (1), 329. 10.1186/s12951-025-03352-8 40319325 PMC12049023

[B54] Meng W. He C. Hao Y. Wang L. Li L. Zhu G. (2020). Prospects and challenges of extracellular vesicle-based drug delivery system: considering cell source. Drug Deliv. 27 (1), 585–598. 10.1080/10717544.2020.1748758 32264719 PMC7178886

[B55] Millar N. L. Silbernagel K. G. Thorborg K. Kirwan P. D. Galatz L. M. Abrams G. D. (2021). Tendinopathy. Nat. Rev. Dis. Prim. 7 (1), 1. 10.1038/s41572-020-00234-1 33414454

[B56] Ren K. (2019). Exosomes in perspective: a potential surrogate for stem cell therapy. Odontology 107 (3), 271–284. 10.1007/s10266-018-0395-9 30324571 PMC6465182

[B57] Rezaie J. Feghhi M. Etemadi T. (2022). A review on exosomes application in clinical trials: perspective, questions, and challenges. Cell Commun. Signal 20 (1), 145. 10.1186/s12964-022-00959-4 36123730 PMC9483361

[B58] Rui Y. F. Lui P. P. Li G. Fu S. C. Lee Y. W. Chan K. M. (2010). Isolation and characterization of multipotent rat tendon-derived stem cells. Tissue Eng. Part A 16 (5), 1549–1558. 10.1089/ten.TEA.2009.0529 20001227

[B59] Rui Y. F. Lui P. P. Wong Y. M. Tan Q. Chan K. M. (2013). Altered fate of tendon-derived stem cells isolated from a failed tendon-healing animal model of tendinopathy. Stem Cells Dev. 22 (7), 1076–1085. 10.1089/scd.2012.0555 23106341 PMC3608022

[B60] Serrano D. R. Juste F. Anaya B. J. Ramirez B. I. Sánchez-Guirales S. A. Quispillo J. M. (2025). Exosome-based drug delivery: a next-generation platform for cancer, infection, neurological and immunological diseases, gene therapy and regenerative medicine. Pharmaceutics 17 (10), 1336. 10.3390/pharmaceutics17101336 41155971 PMC12567338

[B61] Sgaglione J. Neufeld E. V. Swami P. Grande D. A. (2025). Clinical status of exosomes: a review. HSS J., 21 (4), 445–453. 10.1177/15563316251362179 PMC12357840 40832476

[B62] Sharma P. Maffulli N. (2005). Tendon injury and tendinopathy: healing and repair. J. Bone Jt. Surg. Am. 87 (1), 187–202. 10.2106/JBJS.D.01850 15634833

[B63] Sisia G. Giudice E. Attanzio A. Briglia M. Piccione G. Trunfio C. (2026). Exosome and miRNA content engagement in the physical exercise response: what is known to date in atheltic horses? Int. J. Mol. Sci. 27 (1), 520. 10.3390/ijms27010520 41516392 PMC12786952

[B64] Song K. Jiang T. Pan P. Yao Y. Jiang Q. (2022). Exosomes from tendon derived stem cells promote tendon repair through miR-144-3p-regulated tenocyte proliferation and migration. Stem Cell Res. Ther. 13 (1), 80. 10.1186/s13287-022-02723-4 35197108 PMC8867681

[B65] Song W. Ma Z. Wang X. Wang Y. Wu D. Wang C. (2023). Macroporous granular hydrogels functionalized with aligned architecture and small extracellular vesicles stimulate osteoporotic tendon-to-bone healing. Adv. Sci. (Weinh) 10 (34), e2304090. 10.1002/advs.202304090 37867219 PMC10700691

[B66] Song G. Zeng C. Li J. Liu J. Zhao J. Liu B. (2025). Exosome-based nanomedicines for digestive system tumors therapy. Nanomedicine (Lond) 20 (10), 1167–1180. 10.1080/17435889.2025.2493037 40248953 PMC12068745

[B67] Tan Q. Lui P. P. Rui Y. F. (2012). Effect of *in vitro* passaging on the stem cell-related properties of tendon-derived stem cells-implications in tissue engineering. Stem Cells Dev. 21 (5), 790–800. 10.1089/scd.2011.0160 21627568 PMC3295857

[B68] Tarafder S. Chen E. Jun Y. Kao K. Sim K. H. Back J. (2017). Tendon stem/progenitor cells regulate inflammation in tendon healing *via* JNK and STAT3 signaling. Faseb J. 31 (9), 3991–3998. 10.1096/fj.201700071R 28533328 PMC5572690

[B69] Vafadar A. Maleki M. Ehtiati S. Moghadam H. Z. Savardashtaki A. (2026). Optical biosensors for detection of exosomes. Clin. Chim. Acta 579, 120704. 10.1016/j.cca.2025.120704 41207599

[B70] Verma N. Arora S. (2025). Navigating the global regulatory landscape for exosome-based therapeutics: challenges, strategies, and future directions. Pharmaceutics 17 (8), 990. 10.3390/pharmaceutics17080990 40871013 PMC12389065

[B71] Villani C. Murugan P. George A. (2024). Exosome-laden hydrogels as promising carriers for oral and bone tissue engineering: insight into cell-free drug delivery. Exosome-Laden Hydrogels As Promis. Carriers Oral Bone Tissue Eng. Insight Into Cell-Free Drug Deliv. [J/OL] 25 (20), 11092. 10.3390/ijms252011092 PMC11508290 39456873

[B72] Von Stade D. Meyers M. Johnson J. Schlegel T. Romeo A. Regan D. (2025). Primary human macrophage and tenocyte tendon healing phenotypes changed by exosomes per cell origin. Tissue Eng. Part A 31 (15-16), 1109–1120. 10.1089/ten.tea.2024.0143 39761039

[B73] Wang Y. He G. Guo Y. Tang H. Shi Y. Bian X. (2019). Exosomes from tendon stem cells promote injury tendon healing through balancing synthesis and degradation of the tendon extracellular matrix. J. Cell Mol. Med. 23 (8), 5475–5485. 10.1111/jcmm.14430 31148334 PMC6653097

[B74] Wang Y. Jin S. Luo D. He D. Yu M. Zhu L. (2023a). Prim-O-glucosylcimifugin ameliorates aging-impaired endogenous tendon regeneration by rejuvenating senescent tendon stem/progenitor cells. Bone Res. 11 (1), 54. 10.1038/s41413-023-00288-3 37872152 PMC10593834

[B75] Wang S. Yao Z. Chen L. Li J. Chen S. Fan C. (2023b). Preclinical assessment of IL-1β primed human umbilical cord mesenchymal stem cells for tendon functional repair through TGF-β/IL-10 signaling. Heliyon 9 (11), e21411. 10.1016/j.heliyon.2023.e21411 37954299 PMC10638607

[B76] Wang F. Feng J. Jin A. Shao Y. Shen M. Ma J. (2025). Extracellular vesicles for disease treatment. Int. J. Nanomedicine 20, 3303–3337. 10.2147/ijn.s506456 40125438 PMC11928757

[B77] Wen H. Zhang Q. Tang M. Li Y. Tan H. Fang Y. (2024). Study on injectable chitosan hydrogel with tendon-derived stem cells for enhancing rotator cuff tendon-to-bone healing. Zhongguo Xiu Fu Chong Jian Wai Ke Za Zhi 38 (1), 91–98. 10.7507/1002-1892.202309014 38225847 PMC10796223

[B78] Wessler S. Meisner-Kober N. (2025). On the road: extracellular vesicles in intercellular communication. Cell Commun. Signal 23 (1), 95. 10.1186/s12964-024-01999-8 39966900 PMC11837664

[B79] Wu Y. F. Chen C. Mao W. F. (2024). Identification of periostin positive cell population during the long-term culture of mouse tendon-derived cells in late passage. Stem Cells Dev. 33 (13-14), 376–386. 10.1089/scd.2023.0268 38676599

[B80] Wu Y. Zhang T. Miao Y. Zhang A. Wang S. Li X. (2025). Hypoxia induces HIF-1α activation in tendon stem cells to enhance extracellular vesicle-mediated tendon repair. ACS Appl. Mater. and Interfaces 17 (30), 42637–42657. 10.1021/acsami.5c05369 40675622

[B81] Xiang H. Bao C. Chen Q. Gao Q. Wang N. Gao Q. (2024). Extracellular vesicles (EVs)' journey in recipient cells: from recognition to cargo release. J. Zhejiang Univ. Sci. B 25 (8), 633–655. 10.1631/jzus.b2300566 39155778 PMC11337091

[B82] Xu T. Xu M. Bai J. Lin J. Yu B. Liu Y. (2019). Tenocyte-derived exosomes induce the tenogenic differentiation of mesenchymal stem cells through TGF-β. Cytotechnology 71 (1), 57–65. 10.1007/s10616-018-0264-y 30599073 PMC6368508

[B83] Xu H. T. Zhang H. Shen K. Zhou H. Song H. H. Guo D. M. (2025a). Effects of adipose stem cell-derived exosomes on rat tendon healing and its impact on the periphery neuropeptides expression. Zhonghua Yi Xue Za Zhi 105 (7), 544–553. 10.3760/cma.j.cn112137-20240820-1910 39956629

[B84] Xu Y. Shi X. Lin H. Li S. Zhang Z. Wei F. (2025b). GelMA/HA-NB hydrogel encapsulating adipose-derived chondrogenic exosomes enhances enthesis regeneration in chronic rotator cuff tears. Int. J. Biol. Macromol. 309 (Pt 2), 142800. 10.1016/j.ijbiomac.2025.142800 40185430

[B85] Xu Y. Wu B. Zhang Z. Mai Y. Ling Z. Bian L. (2025c). Exosome-infused DTM-MPs hydrogel scaffold: a smart platform for tendon repair with sustained bioactivity and anti-inflammatory benefits. ACS Nano 20 (1), 1030–1046. 10.1021/acsnano.5c16275 41410245

[B86] Xu Y. Peng T. Liang L. Ming Y. Tang Q. Han W. (2026). Tumor exosome-based drug delivery system targeting ferroptosis and apoptosis for glioblastoma therapy. Colloids Surfaces B Biointerfaces 257, 115180. 10.1016/j.colsurfb.2025.115180 41072330

[B87] Xue Z. Chen Z. Wu T. Li R. Chen C. Liu J. (2022). VEGFA-enriched exosomes from tendon-derived stem cells facilitate tenocyte differentiation, migration, and transition to a fibroblastic phenotype. Biomed. Res. Int. 2022, 8537959. 10.1155/2022/8537959 36119932 PMC9481323

[B88] Yamaguchi N. Horio E. Sonoda J. Yamagishi M. Miyakawa S. Murakami F. (2024). Immortalization of mesenchymal stem cells for application in regenerative medicine and their potential risks of tumorigenesis. Int. J. Mol. Sci. 25 (24), 13562. 10.3390/ijms252413562 39769322 PMC11676347

[B89] Yang J. Tang N. Dai R. Chen J. Li Z. Jiang F. (2026). BMSC-derived exosomal miR-21a-5p ameliorates blood–brain barrier injury and hemorrhagic transformation. Mol. Neurobiol. 63 (1), 351. 10.1007/s12035-025-05650-6 41505036 PMC12783216

[B90] Yu H. Cheng J. Shi W. Ren B. Zhao F. Shi Y. (2020). Bone marrow mesenchymal stem cell-derived exosomes promote tendon regeneration by facilitating the proliferation and migration of endogenous tendon stem/progenitor cells. Acta Biomater. 106, 328–341. 10.1016/j.actbio.2020.01.051 32027991

[B91] Zhang J. Li B. Wang J. H. (2011). The role of engineered tendon matrix in the stemness of tendon stem cells *in vitro* and the promotion of tendon-like tissue formation *in vivo* . Biomaterials 32 (29), 6972–6981. 10.1016/j.biomaterials.2011.05.088 21703682 PMC3148341

[B92] Zhang C. Zhu J. Zhou Y. Thampatty B. P. Wang J. H. C. (2019). Tendon stem/progenitor cells and their interactions with extracellular matrix and mechanical loading. Stem Cells Int. 2019, 3674647. 10.1155/2019/3674647 31737075 PMC6815631

[B93] Zhang M. Liu H. Cui Q. Han P. Yang S. Shi M. (2020). Tendon stem cell-derived exosomes regulate inflammation and promote the high-quality healing of injured tendon. Stem Cell Res. Ther. 11 (1), 402. 10.1186/s13287-020-01918-x 32943109 PMC7499865

[B94] Zhang Z. Li Y. Zhang T. Shi M. Song X. Yang S. (2021a). Hepatocyte growth factor-induced tendon stem cell conditioned medium promotes healing of injured achilles tendon. Front. Cell Dev. Biol. 9, 654084. 10.3389/fcell.2021.654084 33898452 PMC8059769

[B95] Zhang M. Liu H. Shi M. Zhang T. Lu W. Yang S. (2021b). Potential mechanisms of the impact of hepatocyte growth factor gene-modified tendon stem cells on tendon healing. Front. Cell Dev. Biol. 9, 659389. 10.3389/fcell.2021.659389 34222233 PMC8250428

[B96] Zhang S. Shang J. Gu Z. Gu X. Wang F. Hu X. (2023a). Global research trends and hotspots on tendon-derived stem cell: a bibliometric visualization study. Front. Bioeng. Biotechnol. 11, 1327027. 10.3389/fbioe.2023.1327027 38260747 PMC10801434

[B97] Zhang T. Wu Y. Li X. Zhang A. Liu H. Shi M. (2023b). Small extracellular vesicles derived from tendon stem cells promote the healing of injured achilles tendons by regulating miR-145-3p. Acta Biomater. 172, 280–296. 10.1016/j.actbio.2023.10.004 37806377

[B98] Zhang M. Dai G. C. Zhang Y. W. Lu P. P. Wang H. Li Y. J. (2024a). Enhancing osteogenic differentiation of diabetic tendon stem/progenitor cells through hyperoxia: unveiling ROS/HIF-1α signalling axis. J. Cell Mol. Med. 28 (20), e70127. 10.1111/jcmm.70127 39467998 PMC11518821

[B99] Zhang X. Song W. Liu Y. Han K. Wu Y. Cho E. (2024b). Healthy tendon stem cell-derived exosomes promote tendon-to-bone healing of aged chronic rotator cuff tears by breaking the positive-feedback cross-talk between senescent tendon stem cells and macrophages through the modulation of macrophage polarization. Small 20 (31), e2311033. 10.1002/smll.202311033 38459643

[B100] Zhang X. Wu Y. Han K. Fang Z. Cho E. Hu Y. (2024c). 3-Dimensional bioprinting of a tendon stem cell-derived exosomes loaded scaffold to bridge the unrepairable massive rotator cuff tear. Am. J. Sports Med. 52 (9), 2358–2371. 10.1177/03635465241255918 38904220

[B101] Zhang T. Li C. Yu J. Mu D. (2026a). Biomimetic “Tendon Band-Aid” regulates inflammatory microenvironment and tenogenic differentiation for tendinopathy regeneration. J. Nanobiotechnology 24 (1), 109. 10.1186/s12951-025-03956-0 41486250 PMC12871026

[B102] Zhang X. Zhou J. Wu J. Yang P. Yuan G. (2026b). Experimental study on the inhibitory effect of eupatilin on osteosarcoma by the NBR2/miR-129-5p/FKBP11 regulatory axis. Ann. Surg. Oncol. 33 (2), 1728–1738. 10.1245/s10434-025-18481-5 41057744

[B103] Zhou Z.-Y. Li Z.-B. Shi N.-S. Feng S. S. Han Y. R. Liu M. T. (2026). Metal-polyphenol network-engineered mesenchymal stem cell-derived exosome mimetics mediate inflammatory/immune regulation for enhanced periodontal tissue regeneration. Biomaterials 327, 123696. 10.1016/j.biomaterials.2025.123696 41005081

[B104] Zou Y. Zhou Y. Li G. Dong Y. Hu S. (2025). Clinical applications of extracellular vesicles: recent advances and emerging trends. Front. Bioeng. Biotechnol. 13, 1671963. 10.3389/fbioe.2025.1671963 41220804 PMC12598036

